# Phage-Mediated Control of *Flavobacterium psychrophilum* in Aquaculture: *In vivo* Experiments to Compare Delivery Methods

**DOI:** 10.3389/fmicb.2021.628309

**Published:** 2021-03-08

**Authors:** Valentina Laura Donati, Inger Dalsgaard, Krister Sundell, Daniel Castillo, Mériem Er-Rafik, Jason Clark, Tom Wiklund, Mathias Middelboe, Lone Madsen

**Affiliations:** ^1^Unit for Fish and Shellfish Diseases, National Institute of Aquatic Resources, Technical University of Denmark, Kgs. Lyngby, Denmark; ^2^Laboratory of Aquatic Pathobiology, Environmental and Marine Biology, Åbo Akademi University, Turku, Finland; ^3^Marine Biological Section, Department of Biology, University of Copenhagen, Helsingør, Denmark; ^4^National Centre for Nano Fabrication and Characterization, Technical University of Denmark, Kgs. Lyngby, Denmark; ^5^Fixed Phage Ltd., Glasgow, United Kingdom

**Keywords:** *Flavobacterium psychrophilum*, rainbow trout fry syndrome (RTFS), rainbow trout fry (*Oncorhynchus mykiss*), phage-therapy, bacteriophages

## Abstract

Phage-based approaches have gained increasing interest as sustainable alternative strategies to antibiotic treatment or as prophylactic measures against disease outbreaks in aquaculture. The potential of three methods (oral, bath, and injection) for delivering a two-component phage mixture to rainbow trout fry for controlling *Flavobacterium psychrophilum* infections and reduce fish mortality was investigated using bacteriophages FpV4 and FPSV-D22. For the oral administration experiment, bacteriophages were applied on feed pellets by spraying (1.6 × 10^8^ PFU g^–1^) or by irreversible immobilization (8.3 × 10^7^ PFU g^–1^), using the corona discharge technology (Fixed Phage Ltd.). The fish showed normal growth for every group and no mortality was observed prior to infection as well as in control groups during the infection. Constant detection of phages in the intestine (∼10^3^ PFU mg^–1^) and more sporadic occurrence in kidney, spleen, and brain was observed. When fish were exposed to *F. psychrophilum*, no significant effect on fish survival, nor a direct impact on the number of phages in the sampled organs, were detected. Similarly, no significant increase in fish survival was detected when phages were delivered by bath (1^st^ and 2^nd^ bath: ∼10^6^ PFU ml^–1^; 3^rd^ bath: ∼10^5^ PFU ml^–1^). However, when phages FpV4 and FPSV-D22 (1.7 × 10^8^ PFU fish^–1^) were administered by intraperitoneal injection 3 days after the bacterial challenge, the final percent survival observed in the group injected with bacteriophages FpV4 and FPSV-D22 (80.0%) was significantly higher than in the control group (56.7%). The work demonstrates the delivery of phages to fish organs by oral administration, but also suggests that higher phage dosages than the tested ones may be needed on feed pellets to offer fish an adequate protection against *F. psychrophilum* infections.

## Introduction

Phage therapy relies on the bactericidal activity of lytic bacteriophages (also called phages), which infect and kill specific bacterial hosts by lysing infected cells and releasing phage progeny to the environment [Twort, 1915; Roux, 2011; reviewed in Salmond and Fineran (2015); Dion et al. (2020)]. In the aquaculture sector, phage therapy efforts have targeted various pathogenic bacteria, such as *Vibrio spp., Aeromonas spp.*, *Flavobacterium spp.*, *Pseudomonas spp.*, and *Edwardsiella spp.* focusing, e.g., on the isolation and characterization of virulent phages (Kalatzis et al., 2016; Kazimierczak et al., 2019), cocktail formulations (Mateus et al., 2014; Duarte et al., 2018), dose and route of phage administration (Laanto et al., 2015; Almeida et al., 2019). However, despite the many benefits of phage therapy, various challenges have been faced such as the development of phage resistant bacteria, the inefficient delivery of phages in high dosages at the infection site, and the phage clearance activity from the organism mediated by the immune cells [reviewed in Culot et al. (2019); Kowalska et al. (2020)].

*Flavobacterium psychrophilum* (Borg, 1948; Bernardet et al., 1996) is the etiological agent of Rainbow Trout Fry Syndrome (RTFS, fry stage) (Lorenzen et al., 1991) and of Bacterial Coldwater Disease (BCWD, juvenile and adult fish) (Borg, 1960). Rainbow trout (*Oncorhynchus mykiss*, Walbaum) and coho salmon (*Oncorhynchus kisutch*) are the most susceptible salmonid species to this bacterium (Nematollahi et al., 2003). Despite a strong focus on preventive measures, for example good management practises and egg disinfection (Nematollahi et al., 2003; Madsen and Dalsgaard, 2008), antibiotics have been the most extensively used treatment for RTFS worldwide and resistance to their activity has been detected (Bruun et al., 2000, 2003; Schmidt et al., 2000; Izumi and Aranishi, 2004; Kum et al., 2008; Del Cerro et al., 2010; Sundell and Wiklund, 2011). With the isolation of bacteriophages infecting *F. psychrophilum* (Stenholm et al., 2008), the possibility of developing a sustainable alternative approach to the treatment of RTFS has gained more attention. Castillo et al. (2012) studied the application of phages in rainbow trout and Atlantic salmon delivering bacteriophages (10^9^ PFU fish^–1^) by intraperitoneal (IP) injection simultaneously to *F. psychrophilum* (10^8^ CFU fish^–1^). The authors were able to detect a reduction in mortality of fish treated with phages (Castillo et al., 2012). Subsequent studies on the dispersal and survival of *F. psychrophilum* phages in rainbow trout showed that infective *F. psychrophilum* phages were recovered from the internal organs of rainbow trout fry after administration by intraperitoneal injection (with and without the bacteria) (Madsen et al., 2013), by bath or via oral administration (oral intubation or by phage-coated feed) (Christiansen et al., 2014). However, in order to assess the potential of phage-based control of *F. psychrophilum* infections in rainbow trout, combined studies on phage delivery efficiency and fish mortality in challenge experiments are required.

Building on previous work (Madsen et al., 2013; Christiansen et al., 2014), this study brings new insights to the development of a bacteriophage-based treatment for *F. psychrophilum* infections in rainbow trout fry applicable in the field. The work includes oral administration of bacteriophages applied on feed pellets by spraying, or by irreversible immobilization, using corona discharge technology (Fixed Phage Ltd.) (Mattey, 2016, 2018). The immobilization stabilizes phages at room temperature, simplifying delivery and use of phage products (Mattey, 2016, 2018). The use of phage-treated feed could potentially be applied prophylactically in aquaculture facilities to prevent and control bacterial infections and mortalities caused by *F. psychrophilum*. Two additional delivery methods (by bath and by intraperitoneal injection) of the selected purified two-component mix of Danish bacteriophages with a wide host-range among virulent *F. psychrophilum* strains were also included in the study. The aim of the work was to (a) evaluate the effects of the oral administration of phages on healthy and infected fish comparing the two phage application methods on fish pellets (e.g., phage diffusion in internal organs) (Experiment A); (b) assess the effects on fish survival of the oral phage administration during *F. psychrophilum* infections (Experiment A) in comparison to when phages are delivered by repeated bath procedures and by intraperitoneal injection (Experiments B and C). The work demonstrates the delivery of phages to fish organs by oral administration, but also suggests that higher phage dosages than the tested ones may be needed on feed pellets to offer fish an adequate protection against *F. psychrophilum* infections for the application in the field.

## Materials and Methods

### Bacterial Strain

*Flavobacterium psychrophilum* 950106-1/1, a well-characterized Danish strain isolated in 1995 from rainbow trout in a freshwater farm, was selected for the experiments (serotype Fd, virulent) (Madsen and Dalsgaard, 1999, 2000; Dalsgaard and Madsen, 2000; Sundell et al., 2019). *Flavobacterium psychrophilum* FPS-S6, a Swedish strain isolated in 2017 from rainbow trout (serotype Th, virulent), was utilized for the propagation of phage FPSV-D22 since it was the most efficient host for producing high phage titers (Sundell et al., 2019). The bacteria were stored at −80°C in tryptone yeast extract salts medium [TYES: 0.4% tryptone, 0.04% yeast extract, 0.05% CaCl_2_ × 2H_2_O, 0.05% MgSO_4_ × 7H_2_O (pH 7.2)] (Holt et al., 1993) and glycerol (15–20%). For bacteriophage detection and quantification, *F. psychrophilum* 950106-1/1 was inoculated from a −80°C stock into 5 ml TYES broth (referred as TYES-B), incubated at 15°C at 100 rpm for 48–72 h and then streaked on TYES agar (TYES-B with 1.1% agar, referred as TYES-A). After 3–4 days at 15°C, single colonies were picked and inoculated in TYES-B for 48 h. For challenge experiments, *F. psychrophilum* 950106-1/1 was prepared and infection challenge performed as described by Madsen and Dalsgaard (1999). Intraperitoneal (IP) injection was selected as infection method due to its reproducibility when it comes to experimental *F. psychrophilum* infections in rainbow trout (Madsen and Dalsgaard, 1999). According to the established infection dose, appropriate dilutions of the 48-h culture were performed prior to IP injection and CFU were counted before and after infection.

### Bacteriophages

Bacteriophages FpV4 and FPSV-D22 were used in these experiments (Supplementary Table 1). FpV4 (lytic phage belonging to *Podoviridae* family, 90 kb genome) was isolated in 2005 from water with feces samples (Stenholm et al., 2008; Castillo and Middelboe, 2016) and FPSV-D22 (lytic phage belonging to *Siphoviridae* family), isolated in 2017 from fish tissue samples collected at Danish freshwater farms of rainbow trout (Sundell et al., 2019). Both phages were characterized to have a broad host range among *F. psychrophilum* strains [(Stenholm et al., 2008; Castillo et al., 2014) and unpublished data]. High titer solutions of FpV4 and FPSV-D22 were purified and stored in SM buffer [8 mM MgSO_4_, 50 mM Tris–Cl (pH 7.5), 99 mM NaCl, 0.01% gelatin] and glycerol (15%) at −80°C (Stenholm et al., 2008; Sundell et al., 2019). The bacteriophages were observed by transmission electron microscopy (TEM) after negative staining with uranyl acetate (Supplementary Table 1). To make these observations, 5 μl of the phage solution were deposited onto a freshly glow discharged carbon-covered grid. The bacteriophage solution was left for 2 min and the grid was negatively stained with 5 μl uranyl acetate (2% in water) for another minute and finally dried using a filter paper. The grids were observed at 200 kV with a Tecnai G2 (FEI) microscope. Images were acquired with a camera Ultrascan US1000 (Gatan). The concentration of phages FpV4 and FPSV-D22 was 10^9^ PFU ml^–1^ in SM buffer with 0.01% gelatin.

### Preparation and Purification of High-Titer Phage Solutions

Based on previous challenge experiments (data not published and Christiansen, 2014), where we detected an early onset of mortality in fish exposed to crude lysates of phages, we decided to PEG purify phage solutions in order to decrease the concentration of compounds that could be toxic for the fish. Gram negative bacteria produce endotoxins which might induce allergic reactions (Kowalska et al., 2020). For the fish experiments, 1 L of bacterial cultures (OD 600 nm = 0.2) were separately infected with the phages FPSV-D22 and FpV4 at MOI = 1 (*F. psychrophilum* FPS-S6 and 950106-1/1, respectively) and incubated for ∼3 days. The lysed bacterial cultures were centrifuged (9000 × *g*, 10 min, 4°C) and filtered through a 0.2 μm-pore size sterile filter. Then, the phage stocks FPSV-D22 (5.0 × 10^9^ PFU ml^–1^) and FpV4 (3.0 × 10^9^ PFU ml^–1^) were concentrated by adding poly-ethylene glycol 8000 (PEG-8000) and Sodium Chloride (final concentration 10% w/v and 1 M, respectively), followed by incubation at 4°C for 24 h. Subsequently, phage solutions were centrifuged (10,000 × *g*, 30 min, 4°C) and the phage pellet was resuspended in 200 mL of SM buffer (Castillo et al., 2019).

### Fish (Experiment A and B)

Rainbow trout eyed eggs were purchased at a Danish commercial fish farm (officially registered free of bacterial kidney disease and viral diseases as IPN, VHS and IHN). Eggs were disinfected with a iodine-based solution [100 ppm active iodine; 1% Actomar K30 (Desag AF, Uster, Switzerland)], hatched and fish grown at the Unit for Fish and Shellfish Diseases (National Institute of Aquatic Resources, Kgs. Lyngby, Denmark). Fish were initially raised in a recirculation system. When the desired size and weight were reached, fish were transferred to a specific laboratory area used for experimental challenges (flow-through system) and divided randomly in 8-L tanks, each with its own inlet/outlet for water and air-supply. Water temperature was constantly maintained at 13°C.

### Fish (Experiment C)

Rainbow trout fry (1–2 g) were purchased from a commercial fish farm in Finland and kept and reared at the fish facilities of Åbo Akademi University (Turku, Finland) in tanks with flow through of dechlorinated tap water (∼12°C) and continuous aeration.

### Experiment A: Delivery of Phages by Phage-Sprayed and Phage-Immobilized Feed

#### Preparation of Phage Feed

Feed pellets (0.8 mm, BIOMAR A/S, Denmark) from the same batch were treated (by spraying or immobilization) with FpV4/FPSV-D22-cocktail [total phage concentration of 3.3 × 10^9^ ± 6.1 × 10^8^ PFU ml^–1^ (SD, *n* = 3)], which was prepared mixing 1:1 PEG-purified solutions of FpV4 (1.2 × 10^9^ PFU ml^–1^) and of FPSV-D22 (4.9 × 10^9^ PFU ml^–1^). In the case of phage-sprayed feed, 30 ml of PEG-purified phage preparation containing FpV4 and FPSV-D22 were applied per 100 g of feed pellets with the use of a spray-bottle as previously described (Christiansen et al., 2014). The process was performed in a flow bench where the feed pellets were left to dry. Fixed Phage Ltd. produced phage-immobilized feed applying 20 ml of PEG-purified phage preparation per 100 g (Mattey, 2016, 2018). Phage-feed pellets were stored at 5°C before use in the experiments.

#### Detection and Quantification of Bacteriophages on Feed

To verify the presence of bacteriophages on feed, the classical method for phage detection was utilized. Three-hundred microliters of a 48-h old *F. psychrophilum* broth culture (in exponential phase) were mixed with 4 ml of TYES soft agar (0.4% agar) and poured into a TYES-A plate (Stenholm et al., 2008; Madsen et al., 2013). For qualitative detection, feed pellets were spread on the bacterial lawn and plates were incubated at 15°C for 3–4 days. Phages on feed pellets were quantified according to Christiansen et al. (2014) with some modifications. Three replicates of 0.1 g of feed and 2 ml of SM buffer were prepared in 2 ml sterile micro tubes (SARSTEDT AG & Co. KG, Germany) for each feed type. A sterile 5 mm steel bead (Qiagen, Germany) was added to each micro tube and samples were homogenized with a Qiagen TissueLyser II (1 min at 20 Hz; Qiagen, Germany). After storage for 1 h at 5°C, samples were transferred to 15 ml sterile Falcon tubes containing 3 ml of sterile SM buffer and vortexed. Phages were quantified by spotting 5 μl of serial 10-fold dilutions (180 μl of SM buffer and 20 μl of sample) of the homogenized solutions in triplicates on a bacterial lawn (TYES soft agar with 48-h old *F. psychrophilum* culture). Plates were incubated at 15°C for 3–4 days and single plaques were then counted in the preferred dilution to estimate the phage titer per gram of feed pellets (Clokie and Kropinski, 2009; Madsen et al., 2013; Christiansen et al., 2014).

#### Set Up and Infection Method

**FIGURE 1:**
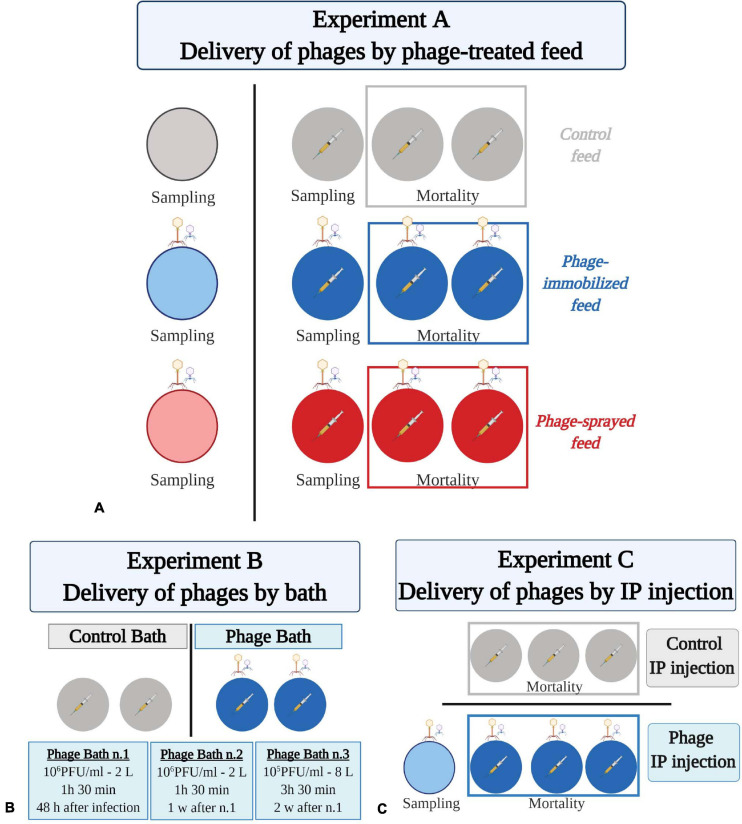
Experimental fish trials to test phage delivery by feed **(A)**, by bath **(B)**, and by intraperitoneal injection **(C)**. **(A)** Eight hundred and ninety-five rainbow trout fry were divided randomly in 12 aquaria (55 fish ± 4) which are represented by colored circles. Fish were fed at 2% of their body weight with either control feed (in gray), phage-immobilized (in blue), or phage-sprayed (in red) feed. Bacteriophage drawings indicate FpV4 and FPSV-D22, which were administered to fish by feed. For each group, three of the four aquaria were challenged with *F. psychrophilum* (1 × 10^4^ CFU fish^–1^; indicated by a syringe with yellow content because of the coloration of the bacteria). Fish in the fourth aquarium were injected with sterile TYES-B as control for the infection. Two of the bacterial challenged aquaria per group were used to follow mortality. The remaining aquaria were dedicated to sampling (five fish at sampling point). **(B)** Hundred and twenty-five rainbow trout fry were divided in four aquaria (circles; 31 fish ± 1 per aquarium) and challenged with *F. psychrophilum* (1 × 10^5^ CFU fish^–1^; syringe with yellow content). Fish in two aquaria were exposed to three rounds of FpV4 and FPSV-D22 phages bath (blue circles; indicated by bacteriophage drawings). Mortality was followed w = week. **(C)** Hundred and twenty rainbow trout (7 g), divided in six aquaria (circles, 20 fish ± 0 per aquarium), were IP challenged with *F. psychrophilum* (1.7 × 10^7^ CFU fish^–1^; syringe with yellow content). Three days later, fish in three aquaria were exposed to phages FpV4 and FPSV-D22 by IP injection (blue circles; indicated by bacteriophage drawings). This aquaria were used to follow mortality. One additional aquarium was included for few sampling where fish were only exposed to the two component phage mix (light blue circle; 15 fish). Created with BioRender.com (the figure was exported under a paid subscription).

The first investigated method of phage treatment was through phage application on feed pellets (by spraying or by using the Fixed Phage Ltd. immobilization technique) (Table 1 and Figure 1A). Rainbow trout fry of 1–2 g were randomly subdivided in 12 × 8 L-aquaria (∼50 fish/aquarium). Fish in four aquaria were fed with phage-sprayed feed; fish in other four aquaria with phage-immobilized feed and fish in the remaining aquaria with control (untreated) feed. All groups were fed at 2% of fish weight per day during the experiment. After a 12-day prophylactic treatment period, fish in three of the four aquaria per feed-type group were exposed to the bacterial pathogen, *F. psychrophilum* 950106-1/1, by IP injection (50 μl, 1 × 10^4^ CFU fish^–1^). Fish in the remaining three aquaria (one aquarium per diet group) were injected with sterile TYES-B (as controls for the infection). Prior to IP injection, fish were anesthetized with 3-aminobenzoic acid ethyl ester (MS-222, Sigma catalog number A-5040). For each feed-type group, two of the infected aquaria were utilized to follow mortality of fish and the two remaining (one infected with the bacterium and one non-infected) were used for live fish sampling during the experiment. Dead and moribund fish were weighed, their length measured, and bacteriological examination of spleen, kidney and brain performed. If possible, internal organs were also collected and stored for phage detection/quantification. During the experiment, several parameters were considered to evaluate the fish health status: feed intake and swimming activity (behavioral observations); fin condition, presence of wounds and coloration (darkening) (external appearance); growth and abnormal mortality (production parameters) (Segner et al., 2019).

**TABLE 1:** Overview of the studied experimental delivery methods.

**Exp.**	**Phage delivery method**	**Administered phage titer**	**Administration time**	**Fish**		**Bacterial infection dose (IP^∗^) (CFU fish**^–1^)
				**Weight (g)**	**Total n. (n. per replicate)**	
**A**	Phage-sprayed feed	1.6 × 10^8^ ± 2.5 × 10^7^ PFU g^–1^ ^*b*^	Continuous feeding started 12 days before IP*	1.9 (±0.7)^*c*^	895 (55 ± 4)^*d*^	1.0 × 10^4^
	Phage-immobilized feed	8.3 × 10^7^ ± 4.8 × 10^7^ PFU g^–1 b^				
	Control feed^*a*^	0 ± 0 PFU g^–1 b^				
**B**	Bath	I. ∼10^6^ PFU ml^–1^ II. ∼10^6^ PFU ml^–1^ III. ∼10^5^ PFU ml^–1^	I. 48 h after IP* (1 h 30 min); II. One (1 h 30 min) and III. two weeks (3 h 30 min) after 1^st^ bath	2–3	125 (31 ± 1) ^d^	1.0 × 10^5^
	Control bath	I. 0 PFU ml^–1^ II. 0 PFU ml^–1^ III. 0 PFU ml^–1^				
**C**	IP injection	1.7 × 10^8^ PFU fish^–1^	3 days after IP*	∼7	120 (20 ± 0)^*d*^	1.7 × 10^7^
	Control IP injection	0 PFU fish^–1^				

*IP, bacterial intraperitoneal injection. ^*a*^Non-treated commercial feed from the same batch as the phage-treated feed types. ^*b*^Average and standard deviation (n = 3). ^*c*^Average weight of fish sampled after 10 days of phage feed prophylaxis (standard deviation in the parenthesis; n = 15). ^*d*^Average number of fish per aquaria and standard deviation in the parenthesis.

#### Fish Sampling

Five fish from each sampling aquaria were sampled randomly at 1, 4, 8, 11, 19, 33, and 56 days post infection. Additionally, five fish were collected from the sampling aquaria not infected with *F. psychrophilum* 1 day before the bacterial challenge. During sampling days, fish were euthanized with an overdose of MS-222. Weight and length of each fish were measured and bacteriological examination of spleen, kidney, and brain performed. To assess the spread of phages in fish, internal organs (spleen, kidney, brain, and the anterior part of the intestine) were collected in pre-weighted 1.5 ml sterile micro tubes (SARSTEDT AG & Co. KG, Germany) containing 300 μl of SM buffer. After the sampling, micro tubes containing fish organs were re-weighted to determine the weight of the organs and 5 μl of chloroform were added under a fume hood to kill any possible bacteria present in the samples. The used phages are not sensitive to chloroform. Fish were not fed for 24 h before sampling.

#### Bacteriological Examination

Using 1 μl sterile inoculation loops, samples from spleen, kidney, and brain were collected for each sampled and moribund/dead fish and streaked on TYES-A. Agar plates were incubated at 15°C from 4 to 5 days up to 4 weeks and *F. psychrophilum* yellow colonies were identified. Randomly chosen yellow colonies were analyzed by MALDI-TOF (Bruker) to confirm that *F. psychrophilum* was the re-isolated bacteria.

#### Bacteriophage Detection and Quantification

Phage detection in the sampled organs was performed as previously described (Madsen et al., 2013; Christiansen et al., 2014). Briefly, chloroform-fixed fish samples were homogenized by vortexing for 20 s and centrifuged for 10 s at 10,000 RPM at 5°C (1 min for intestine samples) to separate chloroform to the bottom of the tube. A spot test method was then performed (Stenholm et al., 2008; Clokie and Kropinski, 2009). For quantification of plaque forming units, 5 μl of undiluted sample were spotted on a freshly prepared bacterial lawn (as described above)in triplicate and incubated at 15°C for 3–4 days. Spots that presented single plaques (from 1 to 30) were counted and the titer of phages per milligram of tissue quantified. In the case of confluent or semi-confluent clearing areas, samples were 10-fold diluted (180 μl of SM buffer and 20 μl of sample) in triplicate and spotted on a bacterial lawn. Plates were incubated at 15°C for 3–4 days, single plaques counted from the preferred dilution and the titer of phages was estimated.

### Experiment B: Delivery of Phages by Bath

Rainbow trout fry of 2–3 g were randomly subdivided in 4 × 8 L-aquaria (∼30 fish/aquarium) (Table 1 and Figure 1B). Fish were fed with commercial feed pellets (0.8 mm, BIOMAR A/S, Denmark) at 2% of fish weight per day during the experiment. Fish in the four aquaria were exposed to the bacterial pathogen, *F. psychrophilum* 950106-1/1, by IP injection (50 μl, 1 × 10^5^ CFU fish^–1^). Based on the results of Experiment A, we decided to increase the infection dose 10 times with the aim of increasing the probability of that bacteria and phages would come into contact with each other. Prior to IP injection, fish were anesthetized with MS-222. The FpV4/FPSV-D22-mix [total phage concentration of 3.3 × 10^9^ ± 6.1 × 10^8^ PFU ml^–1^ (SD, *n* = 3)] was prepared by mixing 1:1 PEG-purified solutions of FpV4 (1.2 × 10^9^ PFU ml^–1^) and of FPSV-D22 (4.9 × 10^9^ PFU ml^–1^). At 48 h post infection, the water in the aquaria was removed and replaced with 2 L of cold tap water containing PEG-purified FpV4 and FPSV-D22 (2 ml of phage mix in 2 L of water – estimated concentration of 3.3 × 10^6^ ± 6.1 × 10^5^ PFU ml^–1^) in two aquaria and 2 L of cold tap water without bacteriophages in the other two aquaria. Fish were bathed in the phage solution for 1 h and 30 min and subsequently, aquaria were filled up and the flow-through water system was re-established. The same procedure was performed 1 week after the first phage bath. One week after the second phage bath, phages were directly administered to the selected aquaria (2 ml of phage mix in 8 L of water – estimated concentration of 8.3 × 10^5^ ± 1.5 × 10^5^ PFU ml^–1^) and the water flow stopped. After 3 h and 30 min, water flow was re-established. The four aquaria were utilized to follow mortality of fish. Dead and moribund fish were weighed, their length measured, and bacteriological examination of spleen, kidney and brain (as for Experiment A) was performed.

### Experiment C: Delivery of Phages Through Intraperitoneal Injection

In experiment C, 120 rainbow trout (∼7 g) were randomly divided in 6 × 150 L-tanks (20 fish/aquarium) and fed with commercial feed pellets (1.2 mm, Rehuraisio, Finland) at 2% of fish weight per day (Table 1 and Figure 1C). Fish in the six aquaria were anesthetized with benzocaine (10%) and exposed to *F. psychrophilum* 950106-1/1, by IP injection (100 μl, 1.7 × 10^7^ CFU fish^–1^). A higher bacterial dose, compared to the previous experiments, was chosen because of the larger fish size. According to Madsen and Dalsgaard (1999), fingerlings have to be challenged with the IP method with an infection dose of 10^7^ CFU fish^–1^ or higher to induce mortalities. At 3 days post infection (dpi), fish in three tanks were exposed to PEG-purified bacteriophages FpV4 and FPSV-D22 by IP injection (100 μl, 1.7 × 10^8^ PFU fish^–1^). Prior to phage exposure, PEG-purified FpV4 (1.2 × 10^9^ PFU ml^–1^) and FPSV-D22 (2.2 × 10^9^ PFU ml^–1^) solutions were mixed 1:1. Fish in the other three tanks were IP injected with sterile SM buffer as controls. Fish mortality was recorded for 21 dpi and, during this period, dead and moribund fish were removed, weighed and bacteriological examination of spleen and kidney performed. A subset of samples were analyzed and verified as *F. psychrophilum* by PCR (Toyama et al., 1994). To ensure delivery of phages in the fish internal organs by this method, fifteen additional rainbow trout were placed in a 150-L aquarium and injected with bacteriophages alone (100 μl, 1.7 × 10^8^ PFU fish^–1^). At 4 and 34 days after exposure, spleen and kidney of five fish were sampled and analyzed for phage detection as described in Experiment A.

### Statistical Analysis

Phage quantification and survival data were analyzed using GraphPad Prism version 8.4.0 for Windows, GraphPad Software, San Diego, CA, United States, www.graphpad.com. For linear regression analysis of phage concentration detected in sampled organs over time, values were first log transformed. In the Kaplan-Meier survival analysis, data from replicate aquaria were merged together [the difference in survival between replicates was ≤20% (Amend, 1981; Midtlyng, 2016)] and comparison of survival curves was performed with the Log-rank (Mantel–Cox) test and the Gehan-Breslow-Wilcoxon test.

## Results

### Phage Delivery by Feed Pellets: Phage-Immobilized and Phage-Sprayed Feed (Experiment A)

In order to evaluate the potential protection conferred by the phage cocktail targeting *F. psychrophilum*, rainbow trout fry (1.5–2 g) were fed either with phage-immobilized (8.3 × 10^7^ ± 2.5 × 10^7^ PFU g^–1^) or phage-sprayed (1.6 × 10^8^ ± 4.8 × 10^7^ PFU g^–1^) feed at 2% of their body weight for 12 days before bacterial challenge (Table 1, Figure 1A and Supplementary Figures 1A,B). Fish growth, abnormal mortalities, feed intake, swimming activity, and external appearance (fin condition and coloration) were monitored during the experiment. Positive growth was detected for all groups (Supplementary Figure 1C and Supplementary Table 2) and no mortalities were observed prior to infection. The addition of phages in either way did not seem to change the taste of the feed for the fish and the fish ate the amount of feed that they were offered (fish not challenged with the bacterium and prior to infection in all groups). Other visual signs of disease conditions, such as destroyed fins, lethargic swimming, color changes, and skin ulceration were not seen prior to infection and in non-challenged groups.

#### Efficiency of Phage Delivery: Percentage of Isolation of *F. psychrophilum* and Its Phages in Fish Organs

**FIGURE 2:**
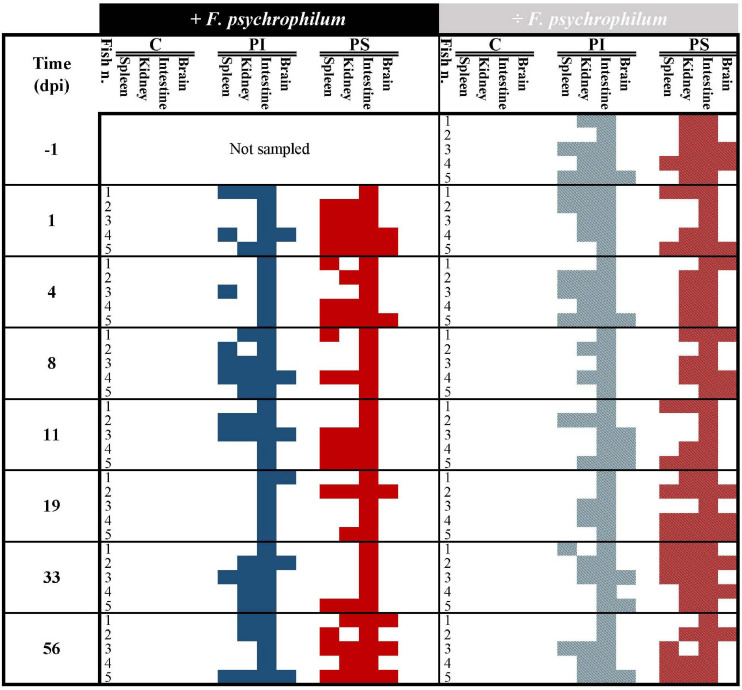
Experiment A. Qualitative detection of bacteriophages in fish organs (spleen, kidney, intestine, and brain) over time in fish fed with phage-immobilized (PI), phage-sprayed (PS), and control (C) feed. Blue and red colors indicate the presence of phages in organs of fish fed with phage-immobilized and phage-sprayed feed, respectively, challenged with *F. psychrophilum* (full color, no pattern) or not (pattern, striped). Absence of phages in the tested organ is indicated by white/blank. Positive detection = presence of one or more plaques in at least one of the technical triplicates. At each sampling point, five fish were sampled in each group except 1 day before the infection where samples were collected only from groups not supposed to be challenged with *F. psychrophilum*. dpi, days post infection.

**FIGURE 3:**
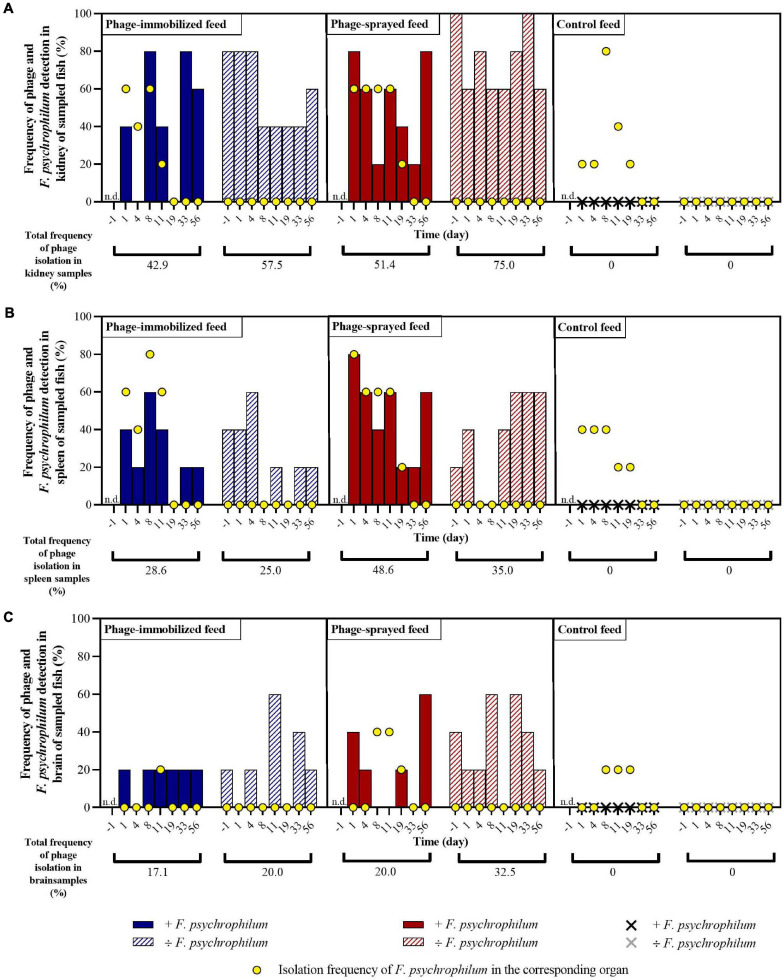
Experiment A. Frequency of detection of bacteriophages and of isolation of *F. psychrophilum* in kidney **(A)**, spleen **(B)**, and brain **(C)** of sampled fish in the different groups over time. No phages were detected in internal organs of fish fed with control feed. Five fish were sampled at each sampling point per group. n.d., not determined. For each organ of each feed group, the total percentage of phages isolation over time is calculated with and without *F. psychrophilum*.

The qualitative detection of bacteriophages in intestine, kidney, spleen, and brain of fish fed with phage immobilized feed, phage sprayed feed, and control feed verified the presence of bacteriophages in treated fish and thereby the delivery of phages through the feed pellets before any manipulation (IP injection with either *F. psychrophilum* or sterile TYES-B) (Figure 2). The results showed that phages were present in the intestine (100% of sampled fish) and in the internal organs of the fish prior to bacterial challenge (100%, 20%, and 40% of kidney, spleen and brain of sampled fish fed with phage-sprayed feed, respectively; 80%, 40%, and 20% of kidney, spleen, and brain of sampled fish fed with phage-immobilized feed, respectively) (Figures 2, 3). Subsequently, we observed the constant presence of bacteriophages in intestines of fish fed with phage-treated feeds during the experiment with more variable occurrence of phages in the other tested organs (Figure 2).

In fish fed with phage-immobilized feed and not exposed to *F. psychrophilum*, bacteriophages were detected in 80% of kidney samples before day 8 and in 40–60% after 8 days post infection (dpi) (Figure 3A). Except for fish sampled 8 dpi, fish challenged with *F. psychrophilum* and fed with phage-immobilized feed (Figure 3A) showed a lower percentage of phages in the fish kidney compared to the controls (0–40% at 1, 4, and 11 dpi; frequency of re-isolation of bacterium = 20–60% until 11 dpi). Subsequently, no bacteria were re-isolated from fish kidney and the frequency of isolation of phages changed from 0% at 19 dpi to 80 and 60% at 33 and 56 dpi, respectively. For spleen samples, we were able to detect phages in 40–60% of fish not challenged with *F. psychrophilum* until 4 dpi and subsequently the percentage of detection dropped to 0–20% (Figure 3B). When fish were also exposed to the bacterium, the percentage of phage detection in spleen was between 20 and 60% until 11 dpi, while *F. psychrophilum* was re-isolated from 40 to 60% of the sampled spleens (Figure 3B). Subsequently, no bacteria were re-isolated and phages were detected in 0–20% of the spleen samples. A lower frequency of phage detection was measured in the brain of fish fed with phage-immobilized feed, whether or not fish were challenged with *F. psychrophilum* (between 0 and 20% except for 11 and 33 dpi where 60 and 40% of sampled brains were positive for phages in non-challenged fish, Figure 3C). *F. psychrophilum* was re-isolated only from the brain of one fish sampled 11 dpi.

Fish fed with phage-sprayed feed (negative to *F. psychrophilum)* were characterized by a consistent phage detection in fish kidney (60–100%; Figure 3A), an increase of the frequency of phage isolation in spleen samples from 20 to 60% during the experiment (Figure 3B) and more variable measurements in the fish brain (20–60%; Figure 3C). When fish were exposed to *F. psychrophilum* (Figures 3A,B), we observed a decrease in phage detection in kidney and spleen samples from 80 to 20% from 1 dpi to 19 and 33 dpi. Subsequently, the frequency of phage detection was increased to 80% for kidney samples (56 dpi) and to 60% for spleen samples (56 dpi). A similar pattern was observed for brain samples (Figure 3C) where our measurements showed an initial decrease in phage detection (from 40 to 20%) with a higher detection frequency at 56 dpi. (60%). For *F. psychrophilum*, we detected high frequencies in spleen and kidney (80% for spleen samples and 60% of kidney samples) in the first days after the bacterial challenge, which decreased during the experiment. In brain samples, *F. psychrophilum* was detected at 8 and 11 dpi (40% of sampled fish).

In fish fed with control feed, the frequency of isolation of the bacteria in kidney samples (Figure 3A) shifted from 20% at 1 and 4 dpi to 80, 40, and 20% at 8, 11, and 19 dpi, respectively. *F. psychrophilum* was re-isolated from 40% of spleen samples until 8 dpi and to 20% at 11 and 19 dpi (Figure 3B). *F. psychrophilum* was only detected in the brain of one fish at 8, 11, and 19 dpi (Figure 3C). No phages were detected in fish fed with control feed and no *F. psychrophilum* was re-isolated from fish that served as negative control for the infection fed in any of the three feed groups (IP with sterile TYES-B) (Figure 3).

#### Efficiency of Phage Delivery in Fish Organs: Quantification

**FIGURE 4:**
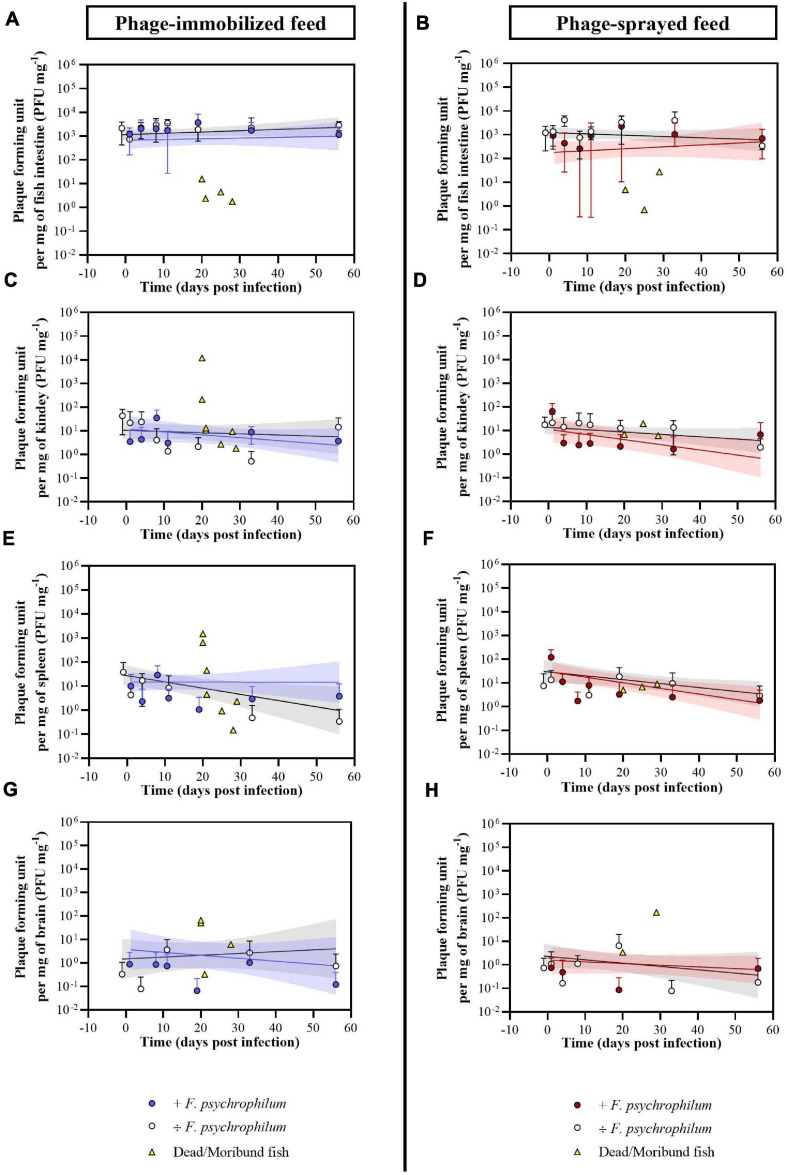
Experiment A. Quantification of bacteriophages FpV4 and FPSV-D22 in intestine **(A,B)**, kidney **(C,D)**, spleen **(E,F)**, and brain **(G,H)** of fish fed with phage-immobilized **(A,C,E,G)** and phage-sprayed **(B,D,F,H)** feed. Values represent the average of five biological replicates per time point and error bars the standard deviation. Concentration of phages in the organs of dead fish (dead because of *F. psychrophilum* infection) is included in the graphs for the corresponding organ and feed type (each symbol in yellow represents a single fish). Simple linear regression lines were calculated from the log-transformed PFU over time and 95% confidence bands are also presented.

After 12 days of phage prophylactic administration, the concentration of phages detected in the intestine of fish was 2.2 × 10^3^ ± 1.7 × 10^3^ PFU mg^–1^ and 1.2 × 10^3^ ± 1.0 × 10^3^ PFU mg^–1^ (day −1) in fish fed with phage-immobilized and phage-sprayed feed, respectively, and these concentrations were maintained over time when fish were not challenged with the bacterium (Figures 4A,B). In fish exposed to *F. psychrophilum*, the intestinal phage concentration was also maintained in fish fed with phage-immobilized feed even if we observed a larger variation (SD) among the biological replicates 11 dpi (1.7 × 10^3^ ± 1.7 × 10^3^ PFU mg^–1^) (Figure 4A). A different situation was observed in challenged fish fed with phage-sprayed feed where a decrease in the intestinal phage concentration was detected in the first 8 days after the bacterial challenge. Indeed, the titer of phages per mg of intestine decreased from 9.1 × 10^2^ ± 5.2 × 10^2^ PFU mg^–1^ measured at 1 dpi to 2.6 × 10^2^ ± 3.5 × 10^2^ PFU mg^–1^ 8 dpi. Subsequently, the number of phages detected in the intestines started to rise even if a large variation among the sampled fish was detected at 11 and 19 dpi. Thirty-three days after the infection the intestinal phage titer raised to 1.0 × 10^3^ ± 1.2 × 10^3^ PFU mg^–1^ (Figure 4B).

Bacteriophages were detected in the kidney of 57.5% and 75.0% of non-challenged sampled fish and of 42.9% and 51.4% of challenged sampled fish fed with phage-immobilized and phage-sprayed feed, respectively (Figure 3). In fish fed with phage-immobilized feed (Figure 4C), the titer of phages in the kidney was ∼10^1^ PFU mg^–1^ 1 day before the infection and at 1 and 4 dpi. The titer per mg of kidney decreased in the following days reaching its lowest point at 33 dpi (0.5 ± 0.8 PFU mg^–1^). At the end of the experiment, the concentration of phages in the kidney was restored to 1.4 × 10^1^ ± 2.1 × 10^1^ PFU mg^–1^ (56 dpi). When fish fed with phage-immobilized feed were challenged with the bacterium, the concentration of phages in kidney samples over time was on average similar to the non-challenged fish (the lowest concentration, 4.3 ± 9.7 PFU mg^–1^, was detected 1 dpi and the highest, 3.5 × 10^1^ ± 4.1 × 10^1^ PFU mg^–1^, 8 dpi). For fish fed with phage-sprayed (Figure 4D), the concentration of phages in the kidney was constant over time with values as 1.7 × 10^1^ ± 1.9 × 10^1^ PFU mg^–1^ (1 day before challenge) and 1.3 × 10^1^ ± 1.5 × 10^1^ PFU mg^–1^ (19 dpi). In case of bacterial challenge, the overall concentration of phages per mg of kidney decreased 10 times compared to non-challenged fish (e.g., 2.8 ± 4.7 PFU mg^–1^ detected 11 dpi) except for the initial high phage concentration detected 1 dpi (6.4 × 10^1^ ± 7.3 × 10^1^ PFU mg^–1^).

Bacteriophages were detected in the spleen of 25.0% and 35.0% of non-challenged sampled fish and of 28.6% and 48.6% of challenged sampled fish fed with phage-immobilized and phage-sprayed feed, respectively (Figure 3). Quantifying the number of phages per mg of spleen in fish fed with phage-immobilized feed (Figure 4E), we observed a decrease from 3.8 × 10^1^ ± 5.8 × 10^1^ PFU mg^–1^ detected 1 day before the bacterial challenge to 0.5 ± 1.1 PFU mg^–1^ and 0.3 ± 0.8 PFU mg^–1^ measured 33 and 56 dpi, respectively. In fish challenged with the bacteria, the number of phages detected over time was stable with values between 2.9 × 10^1^ ± 4.1 × 10^1^ PFU mg^–1^ (8 dpi) and 1.1 ± 2.3 PFU mg^–1^ (19 dpi). In fish fed with phage-sprayed feed (Figure 4F), the concentration of phages per mg of spleen was 1.2 × 10^2^ ± 1.3 × 10^2^ PFU mg^–1^ (+ *F. psychrophilum*) and 1.4 × 10^1^ ± 1.9 × 10^1^ PFU mg^–1^ (÷ *F. psychrophilum*) measured 1 day post infection, and 1.8 ± 3.2 PFU mg^–1^ (+ *F. psychrophilum*) and 2.9 ± 4.6 PFU mg^–1^ (÷ *F. psychrophilum*) 56 dpi.

The number of phages per mg of brain was also quantified (Figures 4G,H), although the percentage of detection over time was low (20.0% and 32.5% of non-challenged sampled fish and 17.1% and 20.0% of challenged sampled fish fed with phage-immobilized and phage-sprayed feed, respectively – Figure 3). In fish fed with phage-immobilized feed (Figure 4G), the concentration of phages per mg of brain was 0.9 ± 1.9 PFU mg^–1^ (+ *F. psychrophilum*) and 0.0 ± 0.0 PFU mg^–1^ (÷ *F. psychrophilum*) measured 1 day post infection, and 0.1 ± 0.3 PFU mg^–1^ (+ *F. psychrophilum*) and 0.7 ± 1.6 PFU mg^–1^ (÷ *F. psychrophilum*) 56 dpi. In Figure 4H, the quantification of phages in the brain of fish fed with phage-sprayed feed is presented. We detected 0.8 ± 1.2 PFU mg^–1^ (+ *F. psychrophilum*) and 1.1 ± 2.4 PFU mg^–1^ (÷ *F. psychrophilum*) measured 1 day post infection, and 0.7 ± 1.2 PFU mg^–1^ (+ *F. psychrophilum*) and 0.2 ± 0.4 PFU mg^–1^ (÷ *F. psychrophilum*) 56 dpi. The phage propagation rates in fish organs over time, calculated from linear regression analysis, are presented in Supplementary Table 3.

Eight and four randomly chosen dead fish were sampled for phage analysis in the groups fed with phage-immobilized and phage-sprayed feed, respectively (Figure 4 and Supplementary Table 4). For fish fed with phage-immobilized feed, phage concentration was between 0.0 and 15.6 PFU mg^–1^ in the intestine, 0.0 and 45.3 PFU mg^–1^ in the spleen, 0.0 and 13.3 PFU mg^–1^ in the kidney, and 0.0 and 65.9 PFU mg^–1^ in the brain. Only two fish (n. 1 and n. 2; Supplementary Table 4) showed a higher concentration of phages in kidney and spleen compared to the others (1.2 × 10^4^ and 210.5 PFU mg^–1^ in kidney samples; 1.3 × 10^3^ and 641.9 PFU mg^–1^ in spleen samples). A similar situation was observed for fish fed with phage-sprayed feed where we detected between 0.0 and 27.5 PFU mg^–1^ of intestine, 0.0 and 74.2 PFU mg^–1^ of spleen, 0.0 and 77.8 PFU mg^–1^ of kidney, and 0.0 and 170.8 PFU mg^–1^ of brain. In the control feed group, only one dead fish was sampled for phage analysis and no phages could be detected.

### Effect of Phage Delivery on Fish Survival (Experiments A, B and C)

**FIGURE 5:**
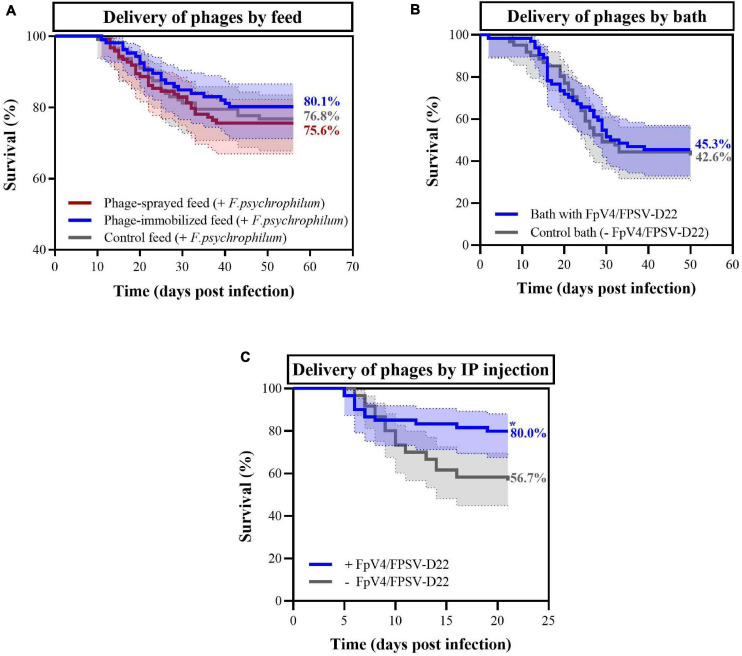
Percent survival observed in rainbow trout fry exposed to *F. psychrophilum* and bacteriophages in the three experiments. In **(A)**, survival of fish fed with phage-immobilized feed (blue), phage-sprayed feed (red) feed and control feed (gray) are displayed (Experiment A). No mortality was observed in the control aquaria (data not shown). In **(B)**, survival of fish bathed in phage solution (blue) and in control bath (gray) in Experiment B. In **(C)**, bacteriophages were delivered with IP injections 3 days after the bacterial challenge (blue) while phage control fish were injected with sterile SM buffer (gray) (Experiment C). In all three experiments, moribund and dead fish were positive to *F. psychrophilum*. Final percent survival are presented for each curve in the figures. * = the curves are significantly different. 95% confidence interval is presented for each curve.

To evaluate the protective effect of bacteriophages and to compare the three phage delivery methods, survival of fish was quantified over time in the three experiments (see experimental set-up in Figure 1 and Table 1). In Experiment A where phages were delivered via the feed, fish mortality started around 10 days post infection (dpi) for the three feed-type groups and it was followed until 56 dpi. The final percent survival for fish fed with phage-sprayed, phage-immobilized and control feed was 75.6%, 80.1%, and 76.8%, respectively (Figure 5A), with no significant differences among the curves. No mortality was observed in the non-challenged groups of fish fed with the three feed types. When phages FpV4 and FPSV-D22 were administered by bath (Experiment B – Figure 5B), the final percent survival was 45.3% in the phage bath group and 42.6% in the control group (no significant difference). When phages FpV4 and FPSV-D22 were administered by intraperitoneal injection 3 days after the bacterial challenge (Experiment C), the final percent survival observed in the control group (56.7%) was significantly lower than the survival of the group injected with bacteriophages FpV4 and FPSV-D22 (80.0%) (Figure 5C).

To confirm the presence of phages delivered by IP injection (Experiment C), five fish were sampled in the control aquaria (only phages) 4 and 34 days after the injection. Four days after IP injection, the concentration of phages detected in the spleen and kidney was 3.3 ± 1.5 and 8.2 ± 3.0 PFU mg^–1^, respectively. Thirty-four days post inoculation, phages were detected in the kidney of two fish (0.03 and 0.06 PFU mg^–1^).

## Discussion

Phage-therapy has gained increased attention in aquaculture as a sustainable alternative strategy to antibiotic treatment or as a prophylactic measure against disease outbreaks. In our study, we investigated the potential of different methods for delivering a two-component phage mixture to rainbow trout fry to control *F. psychrophilum* infections and to reduce fish mortality.

### Phage Delivery by Feed Pellets in Aquaculture

Oral administration of phages for systemic circulation in fish using phage-immobilized (produced by corona discharge technology patented by Fixed Phage Ltd. – final concentration of 8.3 × 10^7^ PFU g^–1^ of feed pellets) and phage-sprayed in-house feed pellets (1.6 × 10^8^ PFU g^–1^) provided a constant delivery of phages to the fish, without a negative effect on fish health or growth. The higher phage density in the internal organs obtained using the phage-sprayed feed compared to the one obtained in fish fed with phage-immobilized feed may partly reflect the higher phage concentration on the manually sprayed feed. However, differences in the orientation of phages on the feed pellets and their detachment and infectivity after the corona discharge and spray treatment, respectively, might also affect the efficiency of delivery. Leppänen et al. (2019) recently showed that the antimicrobial efficiency was higher for detached phages compared to the attached ones and that covalently bound phages on carboxylate-treated gold had the lowest infectivity even if this surface was characterized by the highest number of attached phages. The authors suggest that the lost infectivity of covalently bound phages might have been caused by chemical interactions or the improper orientation of the phages on the surface (Leppänen et al., 2019). In our study, it does not seem that the covalently bound phages on feed pellets by the corona discharge method are characterized by a lower infectivity compared to phages applied using a spraying approach, as indicated by a very similar and stable phage number in the fish intestine when fed with the two phage-feed types. It is not clear if the lower phage translocation efficiency in the internal organs in fish fed with phage-immobilized feed compared with the phage-sprayed feed is due to a tighter binding of the phages to the feed pellets, or a lower number of phages attached to the pellets. Nevertheless, the application of phages on feed pellets by the corona discharge method reduces the time for feed preparation (compared to spraying procedures) and enables the stable immobilization of bacteriophages.

The stable concentration of phages detected in the gut after feeding with phage coated pellets indicated a positive gut transit of the selected phages in the gastrointestinal environment, as described previously (Christiansen et al., 2014). However, the detection of phages in the internal organs (spleen, kidney, and brain) was not always possible, suggesting inefficient phage penetration from the gastrointestinal tract into the systemic circulation. In a systematic analysis of 144 relevant human and animal experiments, the oral administration route was described as the least effective among the tested methods in delivering active phages that penetrate into the circulatory system (Dąbrowska, 2019) presumably by the transcytosis mechanism (Barr, 2017; Nguyen et al., 2017). Indeed, even if bacteriophages can be isolated in feces/intestine indicating a positive gut transit, it is not always possible to isolate them from blood/internal organs. However, the dose of administration can significantly contribute to phage ability to pass to the systemic circulation (Dąbrowska, 2019). Thus, a key step in delivering phages via feed is the application of very high initial titers. In a recent study, the delivery of *Edwardsiella tarda* phage ETP-1 bio-encapsulated in *Artemia* nauplii (enriched *Artemia* at 10^11^ PFU ml^–1^) could provide phages with a concentration of 10^4^–10^6^ PFU mg^–1^ of tissue 10 days after the start of the phage feeding in gut, kidney, spleen and liver of zebrafish (Nikapitiya et al., 2020). Interestingly, it seemed that phage penetration from the gut into the circulatory system was very efficient and only a 10-fold phage decay was observed between the gut and the kidney at the time points examined.

The pH in the gut environment may affect the stability and infectivity of phages as discussed by Madsen et al. (2013) and Christiansen et al. (2014). *In vitro* experiments have shown that the stability of *F. psychrophilum* phages FpV4 and FpV9 were lost completely at pH 3 and in part reduced (a 10 fold reduction over 90 days) at pH 4.5 (Madsen et al., 2013). These pH values resemble the stomach environment (Bucking and Wood, 2009). FpV9 remained stable at pH 6 and 7.5 (typical of intestine environment) (Bucking and Wood, 2009; Madsen et al., 2013). In our experiment, phages were administered together with the feed, thus the stomach pH would likely have been around 4.9 (Bucking and Wood, 2009; Madsen et al., 2013), potentially causing a minor loss in the amount of infective phages in the stomach. However, other factors like the presence of macromolecules or the bacterial microflora in the gut may potentially protect the phages *in vivo* (Dąbrowska, 2019).

Phage decay in the circulatory system has mainly been attributed to the activity of the innate and the adaptive immune systems, with lymphoid organs considered as the main players in phage clearance and de-activation by phagocytosis [reviewed by Dąbrowska (2019); Van Belleghem et al. (2019)]. In fish, the main non-mucosal lymphoid organs are the thymus, the kidney (in adult fish mainly the head kidney or *pronephros*) and the spleen (Secombes and Wang, 2012). In our Experiment A, we detected a stable concentration of infective phages (∼10^1^ PFU mg^–1^) in the kidney of 57.5 and 75.0% of fish fed with phage-immobilized and phage-sprayed feed, respectively (fish were sampled 24 h after feeding; non-infected fish). These results are in line with previous reported delivery efficiencies using oral intubation (Christiansen et al., 2014) and intraperitoneal injection (Madsen et al., 2013) of *F. psychrophilum* phages in rainbow trout fry, where 75% (24 h after delivery) and 100% (up to 72 h after delivery) of sampled fish showed presence of infective phages in the kidney, respectively (Madsen et al., 2013; Christiansen et al., 2014). The constant detection of infective phages in kidney of fry exposed to the treatment may reflect the role of this well-perfused organ in phage clearance from blood in fish (meaning that phages may be constantly delivered but do not accumulate over time because neutralized), unlike in mammals, where the kidneys have different functions and do not seem to be involved in phage clearance (Dąbrowska, 2019).

The lower detection frequency of active phages in spleen samples compared to intestine and kidney samples, i.e., phage detection in 25 and 35% of spleen of fish fed with phage-immobilized and phage-sprayed feed, respectively (non-infected fish) was also in agreement with previous findings of 100–1000 fold decrease in phage concentration from intestine to spleen (Christiansen et al., 2014). In addition, the concentration of phages in the spleen appeared to decrease by 10 fold over time (non-infected fish). It is not clear if this is a result of the in-activation of phages in the spleen, or a consequence of the relatively low phage dose delivered. The spleen, a main secondary lymphoid organ (in fish and in mammals) and a reservoir of disease in fish (Secombes and Wang, 2012), has been suggested to play an important role in phage clearance (Dąbrowska, 2019). Previous experiments in rainbow trout (5 g) where bacteriophages were administered by oral intubation (100 μl of 1 × 10^8^ PFU ml^−1^) showed a complete disappearance of viable phages from the spleen 27 h after phage exposure (Christiansen et al., 2014). Similar results were obtained in mice after the administration of a vibriophage (oral gavage; 100 μl of 1 × 10^8^ PFU ml^−1^), which showed a very high concentration of infective phages in spleen 6 h after the administration, which then decreased over time reaching 10^1^ PFU mg^–1^ of tissue at 24 h (Jaiswal et al., 2014). Other studies, however, have demonstrated the presence of active phages in the spleen for a few days after exposure. However, it is unclear how quickly the phages are inactivated (Dąbrowska, 2019).

The detection of phages in 20.0% and in 32.5% of brain samples of fish fed with phage-immobilized and phage-sprayed feed, respectively, documented that phages FpV4 and FPSV-D22 are likely able to cross the blood-brain barrier. However, the simultaneous collection of blood during brain sampling procedures cannot be excluded. Since the dose and the delivery route have been identified as major conditions influencing the diffusion of phages in the brain (Dąbrowska, 2019), we also believe that the low percentage of detection is linked to the relatively low phage dose administered by feed pellets.

### Phage Delivery Methods and Fish Survival During *F. psychrophilum* Infection

The first indication that the constant delivery of phage FpV9 through feed (1.5 × 10^8^ PFU g^–1^) could provide a decrease in rainbow trout fry (2.5 g) mortality affected by *F. psychrophilum* was provided by Christiansen (2014) (final cumulative mortality of 40% against 53% of control fish). In our Experiment A, we did not observe a similar outcome when phages were delivered orally (phage-sprayed feed: 1.6 × 10^8^ PFU g^–1^; phage-immobilized feed: 8.3 × 10^7^ PFU g^–1^). The lack of a significant beneficial effect on fish survival after bacterial challenge, may reflect a low phage density and a reduced phage-pathogen encounter rate in the infected organs. Similar results were obtained when phages were delivered by bath.

Intraperitoneal injection of bacteriophages has been suggested to be the best route of administration providing a fast and efficient delivery, and is recommended for systemic or localized infections (Dąbrowska, 2019). Phages spread very rapidly in the organs of the fish when delivered with this method but they can also quickly disappear when their target bacteria are not present (IP injection of FpV9 – 10^7^ PFU fish^–1^) (Madsen et al., 2013). Previous experiments demonstrated the potential of using *F. psychrophilum* phages to treat RTFS administered by IP injection (phage 1H and 6H delivered together with *F. psychrophilum*) (Castillo et al., 2012). In our Experiment C, phages delivered in high concentrations (1.7 × 10^8^ PFU fish^–1^) by intraperitoneal injection 3 days after bacterial challenge were able to combat the bacterial infection and reduce mortality. These results support the utilization of the selected phages to control *F. psychrophilum* when delivered in high dosages. Further, delivering both the pathogen and phages by IP injection likely increases the probability of phage-pathogen encounter in the intraperitoneal cavity.

Phage-therapy may be more effective on acute than on chronic infections, as the application of an adequate dose of the right phage/phages during early stages of a bacterial infection (during log-phase growth and before the establishment of biofilm), should efficiently eliminate the pathogen [D’Hérrelle and Smith, 1930; discussed and reviewed by Abedon (2014)]. Thus, the state and abundance of the bacteria in the body is important to ensure phage-host interaction and phage proliferation in the infected organs. For our experiments, we selected the intraperitoneal injection of *F. psychrophilum* as reproducible infection method, although it is considered relatively harsh for the fish and distant from how it would happen in an aquaculture environment (Madsen and Dalsgaard, 1999). With this method, the injected bacteria spread rapidly in the fish organs (peritoneal cavity, spleen, kidney, and brain) in the first 4 days after the infection procedure (Madsen et al., 2013). As expected, we were able to detect *F. psychrophilum* in the internal organs of the fish 1 dpi but no difference in the development of the infection in the three feed-groups was observed (Experiment A). Similar results were obtained by Madsen et al. (2013) where the simultaneous IP injection of phage FpV9 did not reduce the occurrence of *F. psychrophilum* in the organs. When looking at the concentration of phages, we detected a large variation in intestinal phage concentration among sampled fish in the first 10–20 dpi, which was more prominent in fish fed phage-sprayed feed. This might be the result of the bacterial infection, since the reduced feed intake is one of the clinical signs of infected/diseased fish (Nematollahi et al., 2003). The lower concentration of phages detected in the intestine of dead/moribund fish supports this hypothesis. As observed by Madsen et al. (2013), no increase in phage concentration in the kidney in the presence of *F. psychrophilum* was observed. Only few dead fish showed a higher phage concentration indicating phage proliferation in the organ. A similar situation was observed for spleen samples. More markedly for fish fed with phage-sprayed feed, the overall frequency of phage detection was higher when the bacterium was present (48.6% against 35% of control fish). We believe that the undetected phage proliferation in organs containing the bacterium may be a consequence of the relatively low concentration of phages obtained *in situ* by the administration of phage-treated feed pellets (in experiment A). To conclude, our understanding of phage replication *in situ* is limited and so, one should focus on optimizing the delivery of high densities of phages with the aim of maximizing phage concentrations at the site of infection, without relying on the self-replicating properties of the phage (Abedon and Thomas-Abedon, 2010; Abedon, 2014). In addition, the role of poorly mixed environments (biofilm) for phage-host encounter in fish organs should be better understood (Abedon and Thomas-Abedon, 2010; Abedon, 2014).

Finally, when comparing our results on fish survival, it is important to mention that the fish used in Experiment A and B were at the fry stage (2–3 g), whereas larger fish (fingerlings, ∼7 g) were used in experiment C. This is important as higher mortalities from *F. psychrophilum* infections are generally observed in fry population (80%) than larger fish (fingerlings and bigger; 20%) during disease outbreaks in fish farms (Lorenzen, 1994). Differences in fish size and immune status between experiments (Madsen and Dalsgaard, 1999) thus likely explain why the final mean mortality in the fingerlings in Experiment C was lower than for fish in Experiment B, in control groups, despite that they were challenged with the highest bacterial dose.

## Conclusion and Future Perspectives

Even though phage therapy seems very attractive and straightforward, it presents various drawbacks/challenges. In our experiments, we believe that the delivery of bacteriophages applied by Fixed Phage Ltd. technology on feed pellets represents an effective method of delivering a product in the intestine with a feasible application in the field. It also reduces the time-consuming tasks of spraying and drying feed pellets. We believe that the inefficient delivery of phages to the internal organs (i.e., the high loss of infective phages during the delivery process across the intestinal barrier as well as the potential too low phage concentration applied in feed and bath experiments) was the reason for the lack of a beneficial effect on survival of fish challenged with *F. psychrophilum*. The significant increase in fish survival upon IP administration supports the hypothesis that the delivery of higher dosages of phages at the infection site could positively contribute to fish health/recovery, and emphasizes the need for applying higher concentrations of phages on the feed to account of the loss of infective phages during the delivery process. Based on preliminary experiments, we have indications that increasing the number of phages per gram of feed also increases the re-isolation percentage of phages from the fish organs. We therefore think that it is reasonable to suggest that increasing the phage concentration on the feed will lead to increased phage densities in the target organs. However, establishing the relationship between phage doses on the feed and the subsequent concentration in the organs upon delivery is, however, an important next step in the development and optimization of the feed-based delivery.

## Data Availability Statement

The original contributions presented in the study are included in the article/Supplementary Material, further inquiries can be directed to the corresponding author.

## Ethics Statement

The animal study was reviewed and approved by the Animal Experiments Inspectorate of Denmark (Dyreforsøgstilsynet, permission n. 2013-15-2934-00976 until 07-10-2019 and n. 2019-15-0201-00159 from 08-10-2019) (Experiments A and B) and by the National Animal Experiment Board (Eläinkoelautakunta, ELLA) (personal license, under project ESAVI/4225/04.10.07/2017) (Experiment C).

## Author Contributions

VD: planning and execution of experiments A and B including phage-sprayed feed preparation, fish sampling and phage analysis in fish organs, data preparation and analysis, and writing of the manuscript. ID: planning, execution and supervision of experiments A and B, data interpretation, and manuscript preparation. KS and TW: planning and execution of experiment C and contribution to manuscript preparation. DC: production of PEG-purified phage solutions applied on feed pellets and contribution to manuscript preparation. ME-R: TEM imaging of bacteriophages FpV4 and FPSV-D22 and contribution to manuscript preparation. JC: production of phage-immobilized feed and contribution to manuscript preparation. MM: data interpretation, manuscript preparation, and funding acquisition. LM: planning, execution and supervision of fish experiments A and B, data interpretation, and manuscript preparation. All authors have read and approved the final version of the manuscript.

## Conflict of Interest

JC was employed by the company Fixed Phage Ltd. (Glasgow, United Kingdom). The remaining authors declare that the research was conducted in the absence of any commercial or financial relationships that could be construed as a potential conflict of interest.

## References

[B1] Abedon S. T. (2014). Phage therapy: eco-physiological pharmacology. *Scientifica* 2014:581639. 10.1155/2014/581639 25031881 PMC4054669

[B2] Abedon S. T. Thomas-Abedon C. (2010). Phage therapy pharmacology. *Curr. Pharm. Biotechnol.* 11 28–47. 10.2174/138920110790725410 20214606

[B3] Almeida G. M. F. Mäkelä K. Laanto E. Pulkkinen J. Vielma J. Sundberg L. R. (2019). The fate of bacteriophages in recirculating aquaculture systems (RAS)— towards developing phage therapy for RAS. *Antibiotics* 8:192. 10.3390/antibiotics8040192 31652887 PMC6963195

[B4] Amend D. F. (1981). Potency testing of fish vaccines. *Develop. Biol. Standard* 49 447–454.

[B5] Barr J. J. (2017). A bacteriophages journey through the human body. *Immunol. Rev.* 279 106–122. 10.1111/imr.12565 28856733

[B6] Bernardet J. F. Segers P. Vancanneyt M. Berthe F. Kersters K. Vandamme P. (1996). Cutting a gordian knot: emended classification and description of the genus *Flavobacterium*, emended description of the family *Flavobacteriaceae*, and proposal of *Flavobacterium hydatis* nom. nov. (basonym, *Cytophaga aquatilis* Strohl and Tait 1978). *Int. J. Syst. Bacteriol.* 46 128–148. 10.1099/00207713-46-1-128

[B7] Borg A. F. (1948). *Studies on Myxobacteria Associated with Diseases in Salmonid Fishes.* Ph. D. Thesis, University of Washington, Seattle, WA.

[B8] Borg A. F. (1960). Studies on myxobacteria associated with diseases in salmonid fishes. *Wildlife Dis. Ser.* 8 1–85.

[B9] Bruun M. S. Madsen L. Dalsgaard I. (2003). Efficiency of oxytetracycline treatment in rainbow trout experimentally infected with *Flavobacterium psychrophilum* strains having different *in vitro* antibiotic susceptibilities. *Aquaculture* 215 11–20. 10.1016/S0044-8486(01)00897-3

[B10] Bruun M. S. Schmidt A. S. Madsen L. Dalsgaard I. (2000). Antimicrobial resistance patterns in Danish isolates of *Flavobacterium psychrophilum*. *Aquaculture* 187 201–212. 10.1016/S0044-8486(00)00310-0

[B11] Bucking C. Wood C. M. (2009). The effect of postprandial changes in pH along the gastrointestinal tract on the distribution of ions between the solid and fluid phases of chyme in rainbow trout. *Aquac. Nutr.* 15 282–296. 10.1111/j.1365-2095.2008.00593.x

[B12] Castillo D. Andersen N. Kalatzis P. G. Middelboe M. (2019). Large phenotypic and genetic diversity of prophages induced from the fish pathogen *Vibrio anguillarum*. *Viruses* 11:983. 10.3390/v11110983 31653117 PMC6893619

[B13] Castillo D. Christiansen R. H. Espejo R. Middelboe M. (2014). Diversity and geographical distribution of *Flavobacterium psychrophilum* isolates and their phages: patterns of susceptibility to phage infection and phage host range. *Microb. Ecol.* 67 748–757. 10.1007/s00248-014-0375-8 24557506

[B14] Castillo D. Higuera G. Villa M. Middelboe M. Dalsgaard I. Madsen L. (2012). Diversity of *Flavobacterium psychrophilum* and the potential use of its phages for protection against bacterial cold water disease in salmonids. *J. Fish Dis.* 35 193–201. 10.1111/j.1365-2761.2011.01336.x 22324343

[B15] Castillo D. Middelboe M. (2016). Genomic diversity of bacteriophages infecting the fish pathogen *Flavobacterium psychrophilum*. *FEMS Microbiol. Lett.* 363:fnw272. 10.1093/femsle/fnw272 27915247

[B16] Christiansen R. H. (2014). *Phage-Host Interactions in Flavobacterium Psychrophilum and the Potential for Phage Therapy in Aquaculture.* Ph. D. Thesis, University of Copenhagen, Copenhagen.

[B17] Christiansen R. H. Dalsgaard I. Middelboe M. Lauritsen A. H. Madsen L. (2014). Detection and quantification of *Flavobacterium psychrophilum*-specific bacteriophages *in vivo* in rainbow trout upon oral administration: implications for disease control in aquaculture. *Appl. Environ. Microbiol.* 80 7683–7693. 10.1128/AEM.02386-14 25281377 PMC4249249

[B18] Clokie M. R. J. Kropinski A. M. (eds) (2009). *Bacteriophages. Methods and Protocols. Volume 1: Isolation, Characterization, and Interactions.* Totowa, NJ: Humana Press. 10.1007/978-1-60327-164-6

[B19] Culot A. Grosset N. Gautier M. (2019). Overcoming the challenges of phage therapy for industrial aquaculture: a review. *Aquaculture* 513:734423. 10.1016/j.aquaculture.2019.734423

[B20] Dąbrowska K. (2019). Phage therapy: what factors shape phage pharmacokinetics and bioavailability? Systematic and critical review. *Med. Res. Rev.* 39 2000–2025. 10.1002/med.21572 30887551 PMC6767042

[B21] Dalsgaard I. Madsen L. (2000). Bacterial pathogens in rainbow trout, *Oncorhynchus mykiss* (Walbaum), reared at Danish freshwater farms. *J. Fish Dis.* 23 199–209. 10.1046/j.1365-2761.2000.00242.x

[B22] Del Cerro A. Márquez I. Prieto J. M. (2010). Genetic diversity and antimicrobial resistance of *Flavobacterium psychrophilum* isolated from cultured rainbow trout, *Onchorynchus mykiss* (Walbaum), in Spain. *J. Fish Dis.* 33 285–291. 10.1111/j.1365-2761.2009.01120.x 20059636

[B23] D’Hérrelle F. Smith G. H. (1930). *The Bacteriophage and Its Clinical Application.* Springfield, I11: Charles C. Thomas.

[B24] Dion M. B. Oechslin F. Moineau S. (2020). Phage diversity, genomics and phylogeny. *Nat. Rev. Microbiol.* 18 125–138. 10.1038/s41579-019-0311-5 32015529

[B25] Duarte J. Pereira C. Moreirinha C. Salvio R. Lopes A. Wang D. (2018). New insights on phage efficacy to control *Aeromonas salmonicida* in aquaculture systems: an *in vitro* preliminary study. *Aquaculture* 495 970–982. 10.1016/j.aquaculture.2018.07.002

[B26] Holt R. A. Rohovec J. S. Fryer J. L. (1993). “Bacterial cold-water disease,” in *Bacterial Diseases of Fish*, eds Inglis N. R. Roberts V. Bromage R. J. (Sydney, NSW: Halsted Press), 3–22.

[B27] Izumi S. Aranishi F. (2004). Relationship between *gyr*A mutations and quinolone resistance in *Flavobacterium psychrophilum* isolates. *Appl. Environ. Microbiol.* 70 3968–3972. 10.1128/AEM.70.7.3968-3972.2004 15240271 PMC444768

[B28] Jaiswal A. Koley H. Mitra S. Saha D. R. Sarkar B. (2014). Comparative analysis of different oral approaches to treat *Vibrio cholerae* infection in adult mice. *Int. J. Med. Microbiol.* 304 422–430. 10.1016/j.ijmm.2014.02.007 24656386

[B29] Kalatzis P. G. Bastías R. Kokkari C. Katharios P. (2016). Isolation and characterization of two lytic bacteriophages, φSt2 and φGrn1; phage therapy application for biological control of *Vibrio alginolyticus* in aquaculture live feeds. *PLoS One* 11:e0151101. 10.1371/journal.pone.0151101 26950336 PMC4780772

[B30] Kazimierczak J. Wójcik E. A. Witaszewska J. Guziński A. Górecka E. Stańczyk M. (2019). Complete genome sequences of *Aeromonas* and *Pseudomonas* phages as a supportive tool for development of antibacterial treatment in aquaculture. *Virol. J.* 16:4. 10.1186/s12985-018-1113-5 30621713 PMC6325676

[B31] Kowalska J. D. Kazimierczak J. Sowińska P. M. Wójcik E. A. Siwicki A. K. Dastych J. (2020). Growing trend of fighting infections in aquaculture environment—opportunities and challenges of phage therapy. *Antibiotics* 9:301. 10.3390/antibiotics9060301 32512805 PMC7345527

[B32] Kum C. Kirkan S. Sekkin S. Akar F. Boyacioglu M. (2008). Comparison of *in vitro* antimicrobial susceptibility in *Flavobacterium psychrophilum* isolated from rainbow trout fry. *J. Aquat. Anim. Health* 20 245–251. 10.1577/H07-040.1 19306614

[B33] Laanto E. Bamford J. K. H. Ravantti J. J. Sundberg L. R. (2015). The use of phage FCL-2 as an alternative to chemotherapy against columnaris disease in aquaculture. *Front. Microbiol.* 6:829. 10.3389/fmicb.2015.00829 26347722 PMC4541368

[B34] Leppänen M. Maasilta I. J. Sundberg L. R. (2019). Antibacterial efficiency of surface-immobilized *Flavobacterium*-infecting bacteriophage. *ACS Appl. Bio Mater.* 2 4720–4727. 10.1021/acsabm.9b00242 35021472

[B35] Lorenzen E. (1994). *Studies on Flexibacter Psychrophilus in Relation to Rainbow Trout Fry Syndrome (RTFS).* Ph. D. Thesis, National Veterinary Laboratory & Royal Veterinary and Agricultural University, Århus.

[B36] Lorenzen E. Dalsgaard I. From J. Hansen E. M. Hørlyck V. Korsholm H. (1991). Preliminary investigation of fry mortality syndrome in rainbow trout. *Bull. Eur. Ass. Fish Pathol.* 11 77–79.

[B37] Madsen L. Bertelsen S. K. Dalsgaard I. Middelboe M. (2013). Dispersal and survival of *Flavobacterium psychrophilum* phages *in vivo* in rainbow trout and *in vitro* under laboratory conditions: implications for their use in phage therapy. *Appl. Environ. Microbiol.* 79 4853–4861. 10.1128/AEM.00509-13 23747702 PMC3754709

[B38] Madsen L. Dalsgaard I. (1999). Reproducible methods for experimental infection with *Flavobacterium psychrophilum* in rainbow trout *Oncorhynchus mykiss*. *Dis. Aquat. Organ.* 36 169–176. 10.3354/dao036169 10401582

[B39] Madsen L. Dalsgaard I. (2000). Comparative studies of Danish *Flavobacterium psychrophilum* isolates: ribotypes, plasmid profiles, serotypes and virulence. *J. Fish Dis.* 23 211–218. 10.1046/j.1365-2761.2000.00240.x

[B40] Madsen L. Dalsgaard I. (2008). Water recirculation and good management: potential methods to avoid disease outbreaks with *Flavobacterium psychrophilum*. *J. Fish Dis.* 31 799–810. 10.1111/j.1365-2761.2008.00971.x 19238756

[B41] Mateus L. Costa L. Silva Y. J. Pereira C. Cunha A. Almeida A. (2014). Efficiency of phage cocktails in the inactivation of *Vibrio* in aquaculture. *Aquaculture* 42 167–173. 10.1016/j.aquaculture.2014.01.001

[B42] Mattey M. (2016). Treatement of Bacterial Infections in Aquaculture. International Patent Application no. PCT/EP2016/058809. Geneva: World Intellectual Property Organization.

[B43] Mattey M. (2018). Treatment of Bacterial Infections in Aquaculture. U.S. Patent Application no. 15/567,825. Washigton, DC: U.S. Patent and Trademark Office.

[B44] Midtlyng P. J. (2016). “Chapter 6. Methods for measuring efficacy, safety and potency of fish vaccines,” in *Fish Vaccines*, ed. Adams A. (Basel: Springer Basel), 119–141. 10.1007/978-3-0348-0980-1

[B45] Nematollahi A. Decostere A. Pasmans F. Haesebrouck F. (2003). *Flavobacterium psychrophilum* infections in salmonid fish. *J. Fish Dis.* 26 563–574. 10.1046/j.1365-2761.2003.00488.x 14653314

[B46] Nguyen S. Baker K. Padman B. S. Patwa R. Dunstan R. A. Weston T. A. (2017). Bacteriophage transcytosis provides a meachanism to cross epithelial cell layers. *mBio* 8:e01874–17. 10.1128/mBio.01874-17 29162715 PMC5698557

[B47] Nikapitiya C. Dananjaya S. H. S. Edirisinghe S. L. Chandrarathna H. P. S. U. Lee J. (2020). Development of phage delivery by bioencapsulation of *Artemia* nauplii with *Edwardsiella tarda* phage (ETP-1). *Braz. J. Microbiol.* 51:2153–2162. 10.1007/s42770-020-00324-y 32651888 PMC7688814

[B48] Roux M. (2011). On an invisible microbe antagonistic to dysentery bacilli. Note by M. F. d’Herelle, presented by M. Roux. Comptes rendus academie des sciences 1917; 165:373–5. *Bacteriophage* 1 3–5. 10.4161/bact.1.1.14941

[B49] Salmond G. P. C. Fineran P. C. (2015). A century of the phage: past, present and future. *Nat. Rev. Microbiol.* 13 777–786. 10.1038/nrmicro3564 26548913

[B50] Schmidt A. S. Bruun M. S. Dalsgaard I. Pedersen K. Larsen J. L. (2000). Occurrence of antimicrobial resistance in fish-pathogenic and environmental bacteria associated with four Danish rainbow trout farms. *Appl. Environ. Microbiol.* 66 4908–4915. 10.1128/AEM.66.11.4908-4915.2000 11055942 PMC92398

[B51] Secombes C. J. Wang T. (2012). “The innate and adaptive immune system of fish,” in *Infectious Disease in Aquaculture*, ed. Brian A. (Cambridge: Woodhead Publishing Limited), 3–68. 10.1533/9780857095732.1.3

[B52] Segner H. Reiser S. Ruane N. Rösch R. Steinhagen D. Vehanen T. (2019). *Welfare of Fishes in Aquaculture. FAO Fisheries and Aquaculture Circular No. C1189.* Budapest: FAO.

[B53] Stenholm A. R. Dalsgaard I. Middelboe M. (2008). Isolation and characterization of bacteriophages infecting the fish pathogen *Flavobacterium psychrophilum*. *Appl. Environ. Microbiol.* 74 4070–4078. 10.1128/AEM.00428-08 18469131 PMC2446521

[B54] Sundell K. Landor L. Nicolas P. Jørgensen J. Castillo D. Middelboe M. (2019). Phenotypic and genetic predictors of pathogenicity and virulence in *Flavobacterium psychrophilum*. *Front. Microbiol.* 10:1711. 10.3389/fmicb.2019.01711 31396199 PMC6668605

[B55] Sundell K. Wiklund T. (2011). Effect of biofilm formation on antimicrobial tolerance of *Flavobacterium psychrophilum*. *J. Fish Dis.* 34 373–383. 10.1111/j.1365-2761.2011.01250.x 21488905

[B56] Toyama T. Kita-Tsukamoto K. Wakabayashi H. (1994). Identification of *Cytophage psychrophila* by PCR targeted 16S ribosomal RNA. *Fish Pathol.* 29 271–275. 10.3147/jsfp.29.271

[B57] Twort F. W. (1915). An investigation on the nature of ultra-microscopic viruses. *Lancet* 186 1241–1243. 10.1016/S0140-6736(01)20383-3 PMC2170983 20475326

[B58] Van Belleghem J. D. Dąbrowska K. Vaneechoutte M. Barr J. J. Bollyky P. L. (2019). Interactions between bacteriophage, bacteria, and the mammalian immune system. *Viruses* 11:10. 10.3390/v11010010 30585199 PMC6356784

