# Supplementary material for: Phage-Mediated Control of Flavobacterium psychrophilum in Aquaculture: In vivo Experiments to Compare Delivery Methods
Source: Front Microbiol. 2021 Mar 8;12:628309. doi: 10.3389/fmicb.2021.628309 (PMC7983945; doi:10.3389/fmicb.2021.628309)
Supplement: Supplementary file 1 [file Data_Sheet_1.pdf]

Supplementary Material

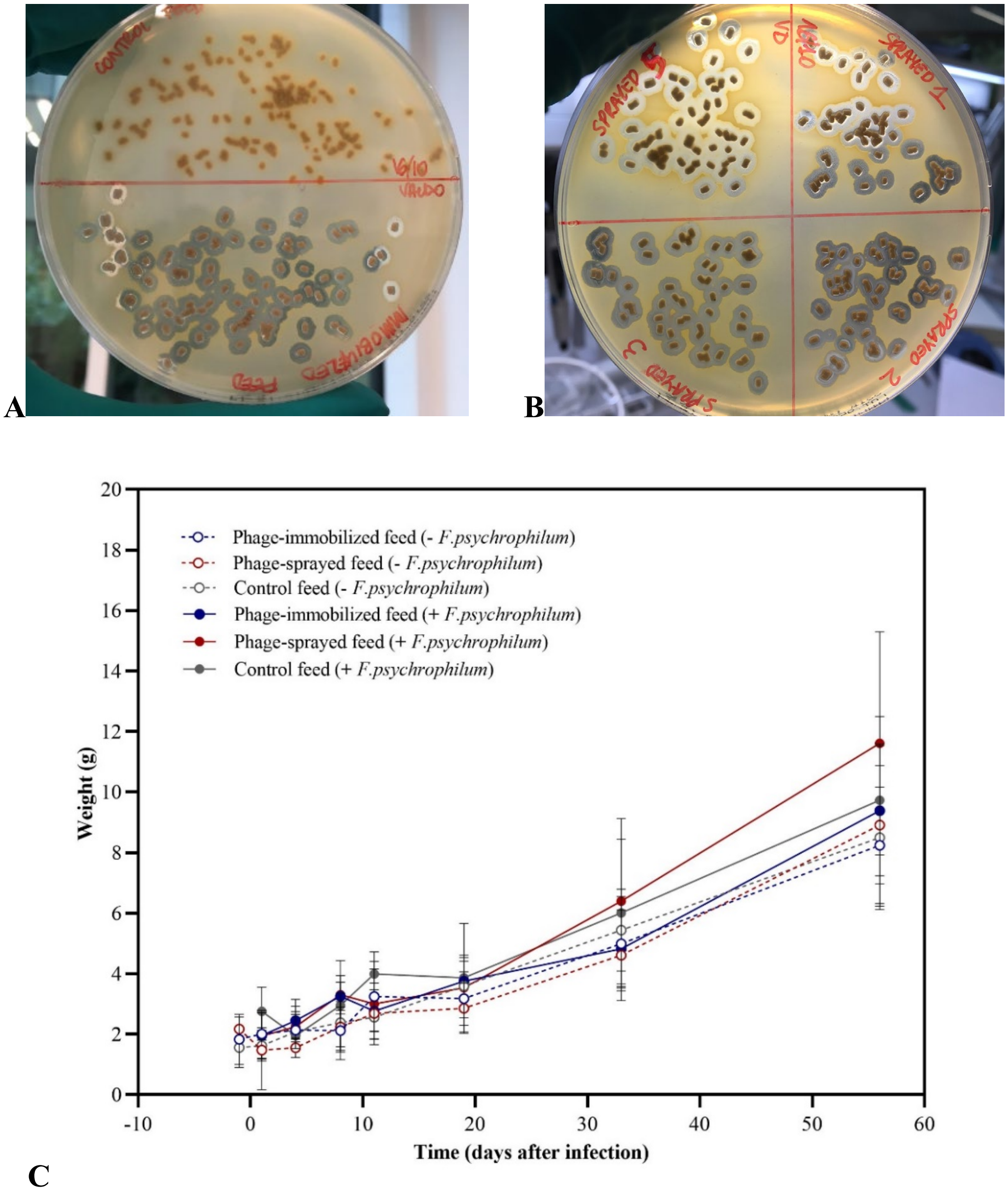

Supplementary Figure 1. Experiment A. Double-layer plaque assay of feed pellets treated with phages (A and B) and growth performance based on weight of fish (C) in the three feed groups with (continuous lines) and without the infection (dashed lines). Phage-immobilized feed (lower panel) and control feed (upper panel) are presented in (**A)** and phage-sprayed feed pellets in (**B) (**the four panels on the plate in B represent four batches of phage-sprayed feed**)**. Clearing areas around feed pellets indicate the presence of bacteriophages. Bacterial lawns were prepared with *F. psychrophilum* 950106-1/1. Pictures were taken after five days of incubation at 15ºC.

Supplementary Table 1. Characteristics of selected virulent phages for experiments A, B and C. Isolation: year and source.

Phage	Isolation	Genome size (kb)	Morphology*	Efficiency of plating (EOP)**	Adsorption constant (ml min^{-1})**	Burst size (PFU ml^{-1})**	Latent period (h)**
			Podoviridae [a]				
**FpV4**[a,b,c]	2005; water with feces [a]	90[a]	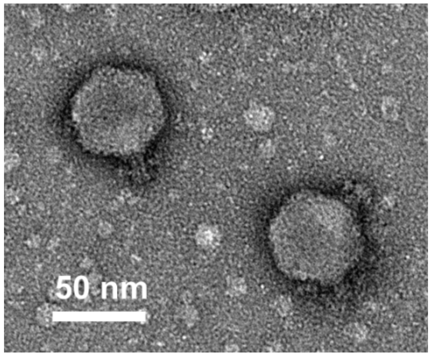	$1.4*10^{-3}$ [c]	$3.4 \times 10^{-10} \pm 7.1 \times 10^{-8}$ [b]	101 ± 7[b]	5.5 ± 0.1[b]
			Siphoviridae (this study)				
**FPSV-D22**[c]	2017; fish tissue [c]	Unpublished data	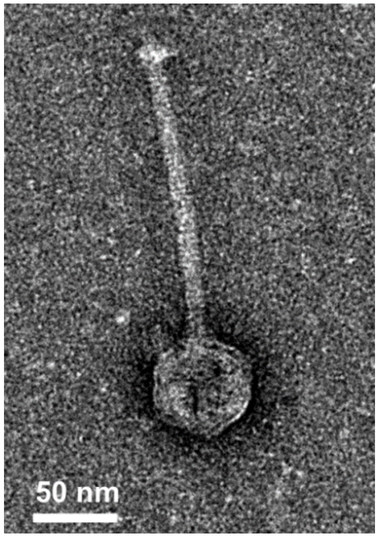	$2.3*10^{-4}$ [c]	Not determined	Not determined	Not determined

[a] Described in Stenholm et al. (2008); [b] Described in Castillo and Middelboe (2016); [c] Described in Sundell et al. (2019).

*The morphology of phage FpV4 was previously observed in Stenholm et al. (2008). In this study, we repeated the TEM imaging of FpV4 together with the recently isolated phage FPSV-D22.

**Host: *Flavobacterium psychrophilum* 950106-1/1.

Supplementary Table 2. Experiment A. Fish growth parameters. Characteristics of fish sampled at day 1 and day 56 post infection for the three feed groups. Mortalities refer to the ones observed in the sampling aquaria. Initial and final fish weight and length data were tested at first with the Shapiro-Wilk test (to evaluate normality) and then compared with ANOVA or Krustal Wallis in case non-normal data distribution. P-values for multiple comparisons were adjusted for Dunnet (normal distribution) or Dunn's (non-normal distribution) corrections. Significantly different P-values (below 0.05) were not observed. The analysis were performed with GraphPad Prism version 8.4.0 for Windows, GraphPad Software, San Diego, California USA, www.graphpad.com. Specific growth rate (SGR) was calculated as follows: SGR(%) = [(ln$Weight_i$ - ln$Weight_f$)/feeding days]*100, where $Weight_i$ and $Weight_f$ indicate the initial and final average fish weight (Egerton et al., 2020).

	**Phage immobilized feed**	**Phage sprayed feed**	**Control feed**	**Phage immobilized feed (+*F. p*)**	**Phage sprayed feed (+*F. p*)**	**Control feed (+*F. p*)**
**Initial weight (g) (mean ±SD; n=5)**	**2.01**±0.21	**1.48**±1.31	**1.62**±0.52	**1.96**±0.76	**1.94**±0.77	**2.76**±0.80
**Initial length (cm) (mean ±SD; n=5)**	**5.54**±0.15	**5.02**±0.11	**5.26**±0.58	**5.52**±0.68	**5.46**±0.77	**6.26**±0.54
**Final weight (g) (mean ±SD; n=5)**	**8.24**±1.92	**8.90**±2.68	**8.50**±2.38	**9.38**±2.16	**11.61**±3.69	**9.73**±2.77
**Final length (cm) (mean ±SD; n=5)**	**8.7**±0.78	**8.98**±0.74	**8.76**±0.89	**9.38**±0.9	**9.94**±0.98	**9.5**±0.94
**Specific growth rate (SGR) (%)**	2.57	3.27	3.02	2.85	3.25	2.29
**Mortalities**	0	0	0	3	4	5

Supplementary Table 3. Experiment A. Phage propagation rate in intestine, kidney, spleen and brain of fish fed with phage-immobilized and phage-sprayed feed (with and without bacterial infection). Values are calculated from linear regression of log transformed PFU over time.

**Phage feed type**	**Bacterial challenge (+ *F. psychrophilum)***	**Tissue**	**Phage propagation**	
			**Rate day^{-1}**	**r^2**
**Phage-immobilized feed**	+	**Intestine**	0.003 (±0.007)	0.005
		**Kidney**	-0.012 (±0.009)	0.11
		**Spleen**	-0.0001 (±0.0002)	0.00003
		**Brain**	-0.012 (±0.011)	0.24
	÷	**Intestine**	0.006 (±0.005)	0.03
		**Kidney**	-0.005 (±0.008)	0.01
		**Spleen**	-0.026 (±0.009)	0.47*
		**Brain**	0.008 (±0.012)	0.06
**Phage-sprayed feed**	+	**Intestine**	0.008 (±0.009)	0.02
		**Kidney**	-0.022 (±0.009)	0.26*
		**Spleen**	-0.024 (±0.007)	0.40*
		**Brain**	-0.008 (±0.006)	0.21
	÷	**Intestine**	-0.005 (±0.005)	0.03
		**Kidney**	-0.010 (±0.006)	0.09
		**Spleen**	-0.017 (±0.007)	0.36*
		**Brain**	-0.014 (±0.010)	0.15

*=the slope is significantly non-zero (p value <0.05)

Supplementary Table 4. Experiment A. Information of dead/moribund fish sampled for phage analysis. Bacteriological examination: a plus sign (+) indicates that *F. psychrophilum* was re-isolated from the corresponding fish organ. dpi = days post infection.

Feed group	Fish n.	Time of event (dpi)	Fish weight (g)	Organs weight (mg)				Bacteriological examination			Presence of phages (PFU mg^{-1} of tissue)			
				Brain	Kidney	Spleen	Intestine	Brain	Kidney	Spleen	Brain	Kidney	Spleen	Intestine
Phage-immobilized feed	1	20	3.21	12.3	13.2	11.3	26.7	+	+	+	50.7	$1.2*10^4$	$1.5*10^3$	0.0
	2	20	2.66	10.2	11.4	8.1	33.0	+	+	+	65.9	210.5	641.9	15.6
	3	28	5.55	18.7	23.2	40.0	16.8		+	+	6.1	9.3	0.2	1.76
	4	21	3.53	17.0	10.0	14.5	17.7	+	+	+	0.0	10.8	4.6	2.4
	5	21	2.70	18.3	12.2	11.1	29.3		+	+	0.3	13.3	45.3	0.0
	6	25	2.46	16.6	12.2	14.4	21.6	+	+	+	0.0	0.0	0.0	4.4
	7	25	2.97	16.0	8.8	8.6	18.6	+	+	+	0.0	2.7	0.9	0.0
	8	29	5.28	16.2	36.6	28.3	45.5	+	+	+	0.0	1.8	2.3	0.0
Phage-sprayed feed	1	15	5.27	19.2	33.9	26.9	20.1		+	+	0.0	0.0	0.0	0.0
	2	20	4.28	14.1	16.0	22.9	16.2	+	+	+	3.4	6.8	4.9	4.8
	3	25	3.51	18.7	21.8	13.3	60.3	+	+	+	0.0	19.5	6.8	0.7
	4	29	2.74	14.5	15.8	9.4	16.6	+	+	+	170.8	6.1	8.9	27.5
Control feed	1	20	3.46	22.5	12.9	11.8	15.4	+	+	+	0.0	0.0	0.0	0.0

References

Castillo, D., and Middelboe, M. (2016). Genomic diversity of bacteriophages infecting the fish pathogen *Flavobacterium psychrophilum*. *FEMS Microbiology Letters* 363. doi:10.1093/femsle/fnw272.

Egerton, S., Wan, A., Murphy, K., Collins, F., Ahern, G., Sugrue, I., et al. (2020). Replacing fishmeal with plant protein in Atlantic salmon (*Salmo salar*) diets by supplementation with fish protein hydrolysate. *Scientific Reports* 10, 4194. doi:10.1038/s41598-020-60325-7.

Stenholm, A. R., Dalsgaard, I., and Middelboe, M. (2008). Isolation and characterization of bacteriophages infecting the fish pathogen *Flavobacterium psychrophilum*. *Applied and Environmental Microbiology* 74, 4070–4078. doi:10.1128/AEM.00428-08.

Sundell, K., Landor, L., Nicolas, P., Jørgensen, J., Castillo, D., Middelboe, M., et al. (2019). Phenotypic and genetic predictors of pathogenicity and virulence in *Flavobacterium psychrophilum. Frontiers in Microbiology* 10, 1–14. doi:10.3389/fmicb.2019.01711.
